# Functional Characterization of IPSC-Derived Brain Cells as a Model for X-Linked Adrenoleukodystrophy

**DOI:** 10.1371/journal.pone.0143238

**Published:** 2015-11-18

**Authors:** Mauhamad Baarine, Mushfiquddin Khan, Avtar Singh, Inderjit Singh

**Affiliations:** Department of Pediatrics, Children's Research Institute, Medical University of South Carolina, Charleston, South Carolina, United States of America; Second Affiliated Hospital, Zhejiang University, CHINA

## Abstract

X-ALD is an inherited neurodegenerative disorder where mutations in the *ABCD1* gene result in clinically diverse phenotypes: the fatal disorder of cerebral childhood ALD (cALD) or a milder disorder of adrenomyeloneuropathy (AMN). The various models used to study the pathobiology of X-ALD disease lack the appropriate presentation for different phenotypes of cALD vs AMN. This study demonstrates that induced pluripotent stem cells (IPSC) derived brain cells astrocytes (Ast), neurons and oligodendrocytes (OLs) express morphological and functional activities of the respective brain cell types. The excessive accumulation of saturated VLCFA, a “hallmark” of X-ALD, was observed in both AMN OLs and cALD OLs with higher levels observed in cALD OLs than AMN OLs. The levels of *ELOVL1* (*ELOVL* Fatty Acid Elongase 1) mRNA parallel the VLCFA load in AMN and cALD OLs. Furthermore, cALD Ast expressed higher levels of proinflammatory cytokines than AMN Ast and control Ast with or without stimulation with lipopolysaccharide. These results document that IPSC-derived Ast and OLs from cALD and AMN fibroblasts mimic the respective biochemical disease phenotypes and thus provide an ideal platform to investigate the mechanism of VLCFA load in cALD OLs and VLCFA-induced inflammatory disease mechanisms of cALD Ast and thus for testing of new therapeutics for AMN and cALD disease of X-ALD.

## Introduction

X-linked adrenoleukodystrophy (X-ALD) is a complex disease where the same mutation in peroxisomal ATP binding cassette superfamily D gene (*ABCD1*) [1,2] can lead to clinically diverse phenotypes even in twin brothers, ranging from the fatal neuroinflammatory disorder of cerebral childhood ALD (cALD) to the adult disorder of adrenomyeloneuropathy (AMN) [3,4]. Biochemical diagnosis is based on the levels of very long chain fatty acids (VLCFA; ≥ C22:0); however, these tests do not distinguish between the different phenotypes of X-ALD. The cALD is characterized by progressive cerebral inflammatory demyelination leading to neurodegeneration and death often before the patient reaches adolescence [2,5–7]. Magnetic resonance imaging (MRI) of the brain is used as a diagnostic tool through detection of demyelination and brain inflammation in cALD. Patients with cALD disease show characteristic white matter lesions [5]. AMN affects adults (third to fourth decade of life) and is characterized by axonopathy (resembling spastic paraparesis or spastic paraplegia) without significant myelin degeneration or neuroinflammation [2,5–7]. However, 35% of AMN patients subsequently develop cerebral demyelination and thus share the same poor prognosis as children with cALD, indicating the complexities in development of different phenotypes of X-ALD disease [2,5–7]. Studies from our laboratory and other groups have reported that VLCFA are catabolized in peroxisomes by the VLCFA β-oxidation enzyme system localized in peroxisomes [8–10]. Subsequent studies from our laboratory and others have reported that VLCFA are imported into peroxisomes [11–13] following conversion of VLCFA-CoA to free fatty acid by thioesterase activity of *ABCD1* [14]. Once inside the peroxisomes, VLCFA are converted to VLCFA-CoA by VLCFA-CoA ligase prior to its catabolism [13,14]. Although the gene abnormality is expressed in all X-ALD cells, different tissues/cells accumulate varying amounts of VLCFA, and the mechanisms for this differentiated load of VLCFA are not well understood. Recent studies [15,16] suggest that in addition to deficient catabolism as a result of loss of *ABCD1* function [9,10,17], the synthesis of VLCFA by *ELOVL1* may contribute to cell specific accumulation of VLCFA [16,18]. Using *ABCD1* silenced Ast (Astrocytes) and OLs (Oligodendrocytes) cell lines, we observed cell specific induction of VLCFA elongation enzyme (*ELOVL1*) in OLs but not Ast, contributing to differential accumulations of VLCFA in OLs and Ast [16]. These observations indicate that cell specific epigenetic factors or modifier genes participate in disease progression of X-ALD. Derangements in *ABCD1* function and *ELOVL1* expression result in pathogenic accumulation of VLCFA in X-ALD. However, the molecular events associated with the transition from a metabolic disease (VLCFA accumulation) to a fatal neuroinflammatory disease in cALD are unclear. The major problem in studying these mechanisms is the unavailability of suitable disease models with cALD and AMN phenotypes. The animal model of ALD disease (*Abcd1* knockout (KO) mice) expresses metabolic disease (the VLCFA accumulation) with signs of oxidative stress without neuroinflammatory disease or brain pathology involvement [19–22]. The brain cell lines presently in use for various investigations encounter limitations in that they carry genetic and epigenetic artifacts of accommodation to tissue culture and/or are derived either from malignant tissues or are genetically modified to drive immortal growth [23]. Various models have been used to study X-ALD, but have not been successful in differentiating between AMN and ALD disease phenotypes [22,24–34]. Since the brain is the primarily affected organ in X-ALD disease, using a human brain cell model would be more effective to study the evolution of phenotype specific disease pathologies.

The ability to generate induced pluripotent stem cells (IPSC) from patient fibroblasts and their further differentiation to specific cell types has become a powerful tool for disease modelling and drug screening [35]. A recent study described IPSC-derived OLs from cALD and AMN fibroblasts with higher VLCFA load in cALD OLs than AMN OLs and AMN neurons [18], indicating the validity of these cell types to study their role in X-ALD disease. Since, both OLs and inflammatory glial cells (Ast and microglia) participate in the pathobiology of X-ALD, we undertook a study to generate IPSC-derived Ast and OLs harbouring cALD and AMN disease phenotypes.

In this manuscript, we report that OLs and Ast from AMN and cALD accumulate VLCFA; however, the levels of saturated VLCFA were higher in cALD cells than in AMN cells. Secondly, cALD OLs have higher VLCFA load compared to AMN OLs. Third, *ELOVL1* gene for synthesis of VLCFA was induced to a higher degree in cALD OLs as compared to AMN OLs, and *ELOVL1* mRNA expression seems to parallel the levels of saturated VLCFA accumulation. Fourth, cALD Ast expresses higher levels (mRNA) of inflammatory mediators (*IL-1β*, *TNFα* and *IL-6*) than AMN Ast. Response to cytokines and/or LPS stimulation was greater in cALD Ast than in AMN Ast. Therefore, these observations describe the generation of IPSC-derived brain cell types (Ast and OLs) responsible for the disease processes of cALD and AMN, providing the rationale for the use of IPSC-derived brain cells to study cALD/AMN disease mechanisms.

## Materials and Methods

### Cell cultures and treatments

Fibroblast cell cultures from healthy individuals (AG01439, male 3 days old), AMN (GM07530, male 26 years old) and cALD (GM04934, male 7 years old with VLCFA abnormality and clinical X-ALD disease) patients were obtained from the Coriell Institute Cell Repositories (Camden, New Jersey). The control human IPSC ATCC-DYR0100 cell line was purchased from ATCC (Manassas, VA). Fibroblasts were cultured in DMEM with 10% FBS, 2mM L-glutamine and 1% penicillin/streptomycin at 37°C with 5% CO_2_.

### Cell reprogramming

Reprogramming of human fibroblasts was carried out using two more genes (c-MYC and KLF4) in addition to four genes (OCT4, SOX2, NANOG and LIN28) described originally [36]. Briefly, fibroblast cells seeded at 0.2 X 10^6^ cells/well of a 6-well plate in fibroblast medium (DMEM + 10% FBS) were transduced with six lentiviral vectors designed to deliver human OCT4, SOX2, c-MYC, KLF4, Nanog and Lin28 cDNA sequences [37]. On the next day, fresh fibroblast media was added to the cells 24 hours after transduction. At 48 hours after transduction the media was changed to half E8 medium and half fibroblast medium. When the cells reached about 60% confluence they were passaged to 10 cm Matrigel-coated plates (one well of a 6-well plate into one 10 cm dish) in E8 medium (StemCell Technologies) and media was replaced daily. Between day 15 and day 30 in culture, individual hiPS clones were manually picked using Leica stereomicroscope. Each hiPS clone was expanded and characterized by immunofluorescence for the expression of Oct4 and Tra-1-60. IPSCs were cultured on a Matrigel (BD-Biosceicnes) coated plate in IPSC medium (mTeSR media from Stemcell technologies, Vancouver, Canada) and media was changed daily until cells were ready for passage.

### In vitro embryonic body formation and characterization

For assessment of pluripotency, an embryoid body assay was performed. For germ layer characterization, the undifferentiated cells were grown onto 10 cm tissue culture dishes, and at 80% confluency were dissociated using EDTA and grown in suspension for three weeks in Petri dishes in IMDM (Lonza) supplemented with 10% FBS (HyClone) and 10 μM ROCK inhibitor (Selleck Chemicals). Rock inhibitor was maintained in the medium for the first 24 hours [37]. After three weeks the embryoid bodies were seeded on Matrigel-coated 6-well plates, fixed and processed for immunofluorescence using the germ layer characterization kit (Millipore). Germ layers were characterized as follows: mesoderm germ for smooth muscle actin (SMA), ectoderm for nestin and endoderm for alpha fetoprotein (AFP).

### Antigenic characterization of IPSC, NPC and differentiated cells

For immunostaining, cells were fixed in 4% paraformaldehyde for 10 minutes, permeabilized with 1.5% Triton X-100 for 10 minutes and blocked in 5% horse serum (Gibco), 1% Triton X-100 in 1 × PBS for 30 minutes at room temperature. For the pluripotency characterization of IPSC, primary antibody staining was performed at 4°C overnight with antibodies against SOX2, OCT4, Lin28, C-MYC, KLF4 and NANOG (Rabbit monoclonal IgG, Cell signaling technology, Danvers, MA) and SSEA4, TRA-1-81 and TRA-1-60 (mouse monoclonal IgG, Cell signaling technology, Danvers, MA). For characterization of neural precursor cells (NPC), cells were incubated overnight with primary antibodies against SOX1 (R and D systems, goat polyclonal), SOX9 (santa cruz, rabbit polyclonal), PAX6 (abcam, rabbit polyclonal) and Nestin (santa cruz, mouse monoclonal). The following antibodies were used to characterize differentiated brain cell types: CNPase (cell signaling, rabbit monoclonal) and myelin basic protein (MBP) (abcam, rabbit polyclonal) for OLs, glial fibrillary acidic protein (GFAP) antibody for Ast (Dako, rabbit polyclonal) and neurofilament (cell signaling, rabbit monoclonal), NeuN (abcam, mouse mmonoclonal) and β-Tubulin (cell signaling, rabbit monoclonal) antibodies for neurons. Cells were then incubated with appropriate secondary antibody conjugated with appropriate fluorescence from Life technologies (Foster City, CA). Nuclei were visualized by staining with 2μg/ml Hoechst 33342 (Sigma Aldrich, St. Louis, MO).

### Differentiation of IPSC to neurons and glial cells (Ast and OLs)

Neural differentiation of IPSC was performed using embryonic body (EB) for respective cell types [38,39]. Briefly, IPSCs were enzymatically detached to single cell suspension using accutase solution. To form standardized EB size, 10000 single IPSC cell suspension per microwell of the aggrewell™ 800 system (stemcell technologies in STEMdiff™), were cultured with neural induction medium (NIM) for five days with daily partial change of NIM medium. Further, EBs were harvested and plated onto poly-L ornithine (Sigma, 20 μg/ml) and laminin (sigma, 10 μg/ml) coated plates for 7 days of culture with daily change of NIM medium. The NPC-containing neural rosette structures (morphological indicator of early neural induction) were selected using the STEMdiff™ neural rosette selection reagent (stemcell technologies). Selected cells were replated onto poly-L ornithine/laminin coated plates and characterized as described above. For terminal differentiation into neurons, NPC were enzymatically dissociated using accutase to a single cell suspension and plated onto poly-L ornithine (Sigma, 20 μg/ml) and laminin (BD Biosciences, 10 μg/ml) coated 100mm petri dishes at concentration of 600000/plate. NPC were cultured in NIM for 2 days, after that media was changed to neural differentiation medium (neurobasal medium (Invitrogen) supplemented with 2% B27 (Invitrogen), 1% glutaMax™-I, and at day 7 of differentiation the medium was supplemented with 0.5mM of dibutyryl cAMP (Sigma) and cultures were maintained for 13 to 16 days. For terminal differentiation into OLs, NPC were enzymatically dissociated by accutase to a single cell suspension and plated on poly-L ornithine (Sigma, 20 μg/ml) and laminin (BD Biosciences, 10 μg/ml) coated 100mm petri dishes at concentration 500000 cells/plate. NPC were cultured in NIM for 2 days, after that the media was changed to a neurobasal medium (Invitrogen) supplemented with 2% B27 (Invitrogen), 1% glutaMax™-I, and 30mg/ml Triiodo-L-Thyronine (T3) (Sigma) and cultures were maintained for 35 to 40 days in this media. For Ast, NPC (500000 cells/plate) plated on a Geltrex™ -coated 100mm petri dishes were cultured for 2 days in NIM medium and then the media was changed to Ast differentiation medium (DMEM (Invitrogen) supplemented with 1% N-2 (Invitrogen), 1% glutaMax™-I, and 1% FBS) and the cultures were maintained for 25 to 30 days. For characterization a parallel set of cultures in an 8 well lab-tek chamber slide were maintained in respective differentiating media for different cell types with partially or complete change of media for neurons and Ast / OLs every 72h respectively, and monitored for degree of differentiation. Purity of cells was determined by counting the number of positive cells for each specific cell type marker in total of 250 cells.

### RNA extraction, cDNA synthesis and gene expression analysis by RT-PCR

Following total RNA extraction using TRIzol (Invitrogen) per the manufacturer's protocol, single-stranded cDNA was synthesized from total RNA by using RT2 First Strand kit and protocol (Qiagen). Briefly, RNA was treated for 5 min at 42°C with gDNA elimination buffer and then incubated with RT cocktail for 5 min at 25°C followed by 45 min at 42°C for cDNA first strand synthesis. The reaction was stopped by heating at 85°C for 5 min. cDNA was synthesized in a 20 μl reaction mixture containing 1–5 μg of total RNA and stored at -20°C until use as described previously [32]. Real time PCR was conducted using Bio-Rad iCycler (iCycler iQ Multi-Color Real Time PCR Detection System; Bio-Rad) real time system. IQ™ SYBR Green Supermix was purchased from Bio-Rad. Primers for human *IL-1β*, *IL-6*, *TNFα*, *GFAP* and *ELOVL1* were purchased from Qiagen. Thermal cycling conditions were as follows: activation of DNA polymerase at 95°C for 10 min, followed by 40 cycles of amplification at 95°C for 30 s and 60°C for 30 s. The normalized expression of a target gene with respect to glyceraldehyde-3-phosphate dehydrogenase or *RPLP0* RNA was computed for all samples using Microsoft Excel data spreadsheet.

### Fatty acid analysis

Total lipids were extracted from control, AMN and X-ALD cells as described previously [26]. Briefly, approximately 5.0 × 10^6^ cells were harvested at 70 to 90% confluence for fatty acids analysis. Fatty Acid Methyl Ester was analyzed by gas chromatography (GC) (Shimadzu chromatograph GC-17A) using a fused silica capillary column (25 M 007 series methyl silicone, 0.25-mm internal diameter, 0.25-μm film thickness) from Quadrex Corporation (Woodbridge, CT) Shimadzu GC with a flame ionization detector. Heptacosanoic acid (C27:0) was used as an internal standard for the quantification of C26:0.

### Galactocerebrosides analysis

Approximately 2.5 × 10^7^ cells from different cell lines were used for lipids extraction as described previously by Singh et al. [40]. Galactocerebrosides were resolved by high performance TLC (LHPK plates from Whatman) as described by Ganser et al. [41] for sphingolipids. Galactocerebroside was quantitated by densitometric scanning using Imaging Densitometer (model GS-800; Bio-Rad), and the software provided with the instrument by the manufacturer.

### Ca^2+^ efflux measurement

The Fluo-4 Direct™ Calcium Assay Kit (Life technologies, NY) was used to measure the Ca^2+^ efflux in a 12 well plate. Briefly, differentiated cells (neurons, OLs and Ast), were incubated for 30 min at 37°C in mix media (culture media and the Fluo-4 solution (1:1)). Glutamate was injected into the media (final concentration 100μM), and 5 seconds later started reading the fluorescence using the microplate reader CLARIOstar from BMG LABTECH (Cary, NC). Fluorescence was followed for 30 seconds.

### Statistical analysis

Statistical analyses were performed on at least three independent experiments. Statistical significance was determined using the non-parametric Mann and Whitney test. Data were expressed as mean ± SD of n determinations. *p* value less than 0.05 was considered statistically significant.

## Results

### Generation and characterization of IPSC from primary healthy, AMN or cALD human fibroblasts

**Fig 1:**
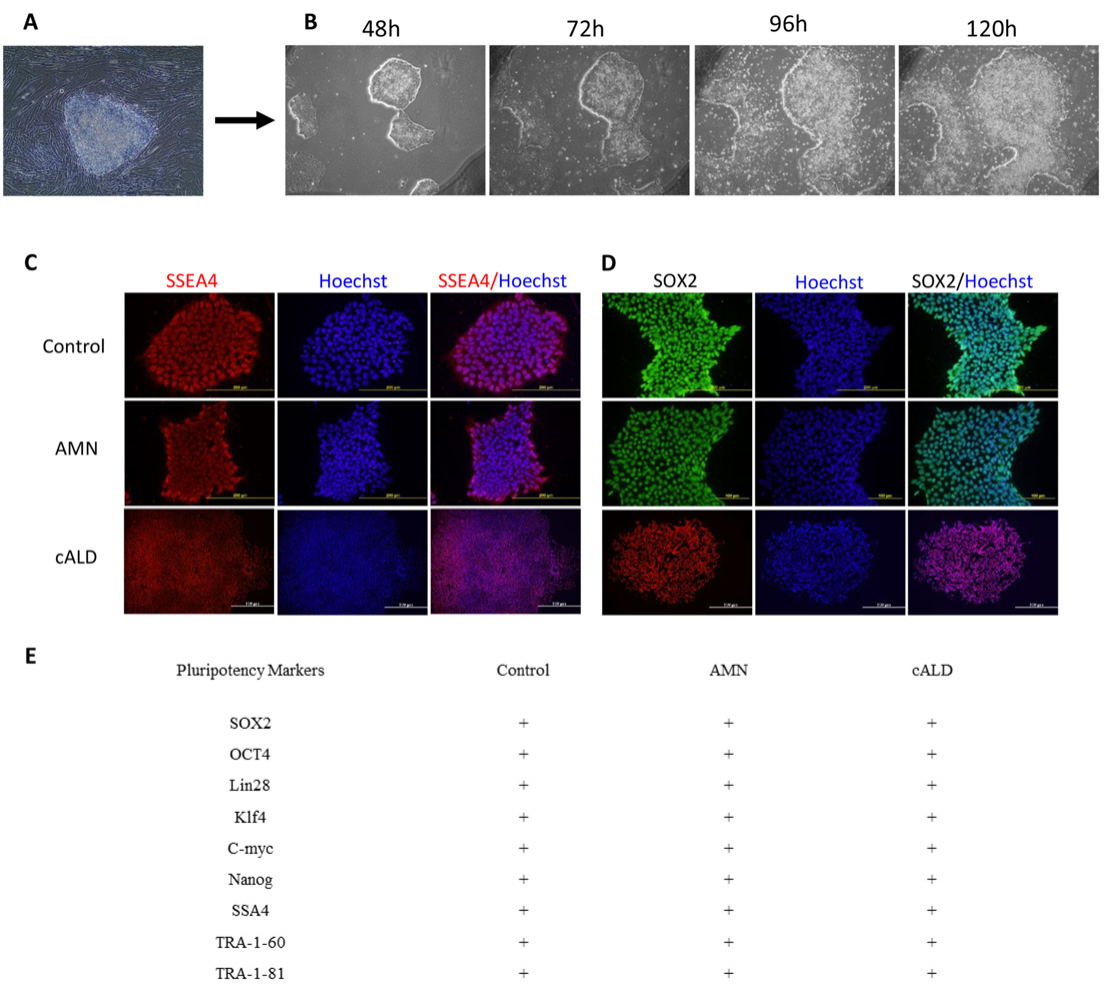
Morphological and specific marker characterization of fibroblast-derived IPSC. (A) Fibroblasts from a male healthy or patient with AMN or cALD disease were transduced with retroviral vectors expressing reprogramming factors OCT4, SOX2, NANOG, LIN28, KLF4, and c-MYC as described under methods. IPSC colony before isolation. (B) A putative control IPSC line was isolated and expanded under feeder-free maintenance medium for human IPSC, colonies growth was observed for 5 days by phase contrast image. (C-D) Control, AMN or cALD IPSC expressed the SOX2 and SSEA4 markers of pluripotency. (E) Summary chart depicts the markers for IPSC lines that were characterized. Scale bars represent 200 μm.

**Fig 2:**
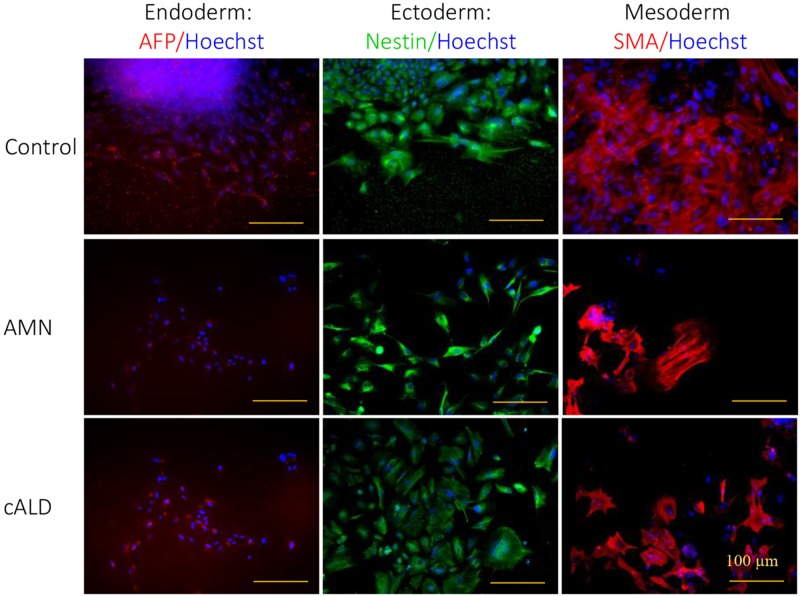
Characterization of pluripotency in the Control, AMN and cALD IPSC. Representative images from different cell types (Control, AMN and cALD) for the 3 embryonic germ layers in vitro. Control, AMN and cALD IPSC lines generated cell types of all three embryonic germ layers (endoderm, AFP; mesoderm, α-SMA; ectoderm, Nestin), as embryoid bodies as decribed under method section (scale bars, 100 μm).

Fig 1 shows the generation of IPSC cells from patient-derived fibroblasts with cALD (G04934, 7 year old male child with clinical disease), AMN (GM07530, 26 year old male) and from control normal human IPSC ATCC-DYR0100 cell line (ATCC, Manassas, VA) and/or the apparently healthy non-fetal tissue the AG01439 (3 DA at sampling, Coriell, New Jersey, USA). Fibroblasts were reprogrammed by retroviral-mediated delivery of six factors as described in the method section (2.2). Selected colonies of IPSC (Fig 1A and 1B), from control (Control IPSC), cALD (cALD IPSC) and AMN (AMN IPSC) fibroblasts were characterized for pluripotent markers and characteristic morphology. Immunofluorescence staining revealed that the colonies (after 5–6 passages) of different ALD disease specific and control cell lines were positive for SOX2 and SSA4 (Fig 1C and 1D). These different cell lines were also positive for other pluripotency markers such as OCT4, Lin28, Klf4, C-myc, Nanog, TRA-1-81, TRA-1-61 (all the introduced factors) (Fig 1E). To examine whether the induced cells had the capacity to differentiate into three germ layers *in vitro*, the embryoid body assay was performed (Fig 2). Differentiation of IPSC through embryoid body (EB) stage resulted in cells typically found in ectoderm (Nestin positive cells), endoderm (AFP positive cells), and mesoderm (SMA positive cells) (Fig 2).

### Generation and characterization of neural precursor cells (NPC)

**Fig 3:**
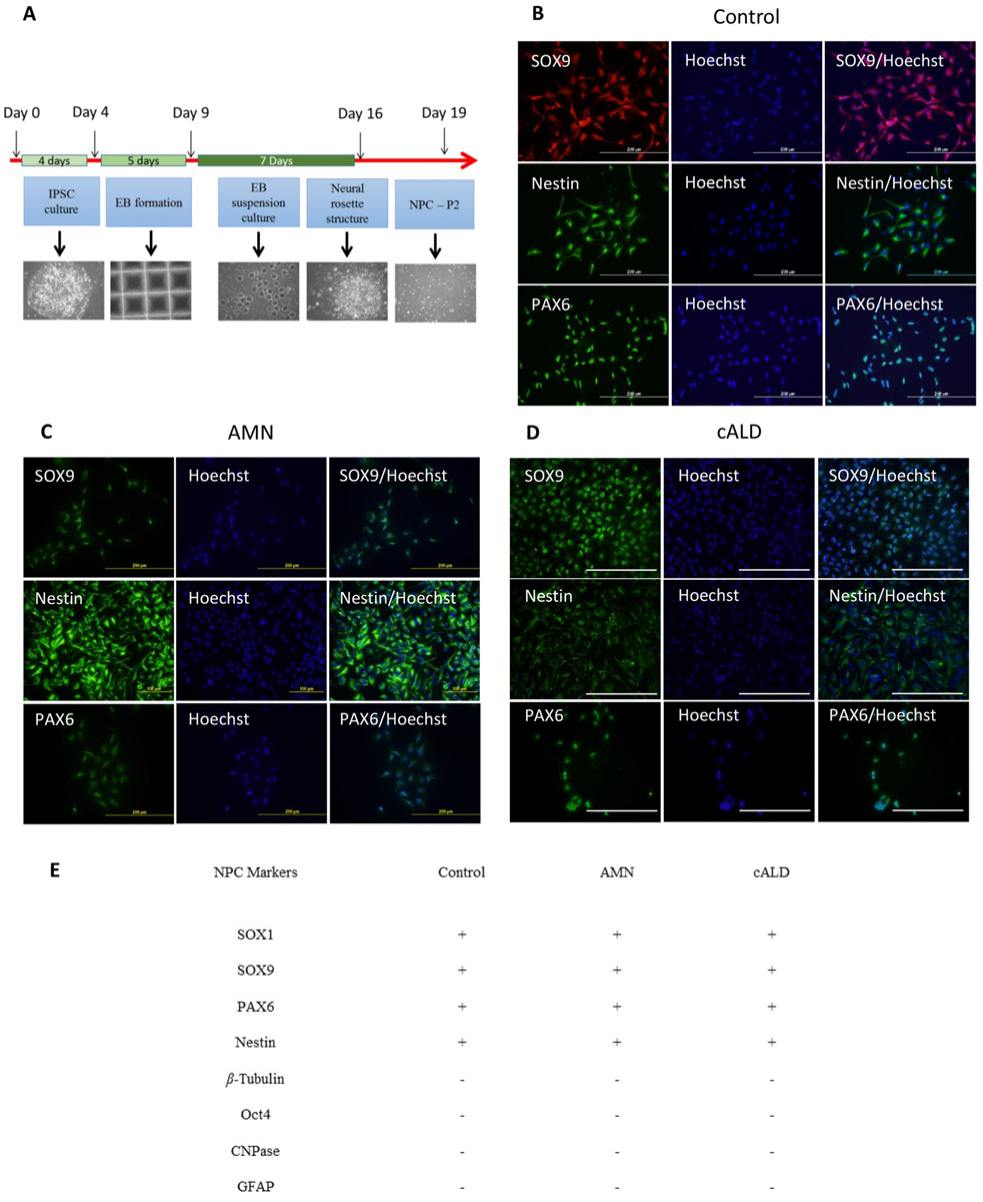
IPSC-derived neural precursor cells differentiation and characterization. (A) Protocol for direct differentiation of human stem cell lines (IPSC) into neural precursor cells. After EBs formation from day 4–9, cells were differentiated as embryoid bodies from day 9–16 in neural induction media where neural rosette structure was selected and plated and second passage cells were analyzed. (B-D) Representative immunostaining results for NPC cultures from different IPSC (Control, AMN and cALD) shows SOX9^+^, PAX6^+^ and Nestin^+^ NPC cells (scale bars, 200 μm). (E) Summary chart of observed positive and negative markers for NPC characterization.

As illustrated in Fig 3A and described in the material and methods section, NPC from IPSC were derived in a four step process. Single cell suspension of IPSC was cultured for 5 days for EBs formation and then for 7 days for EBs culture in PLO/Laminin coated plates for selection of neural rosette structures (morphological indicator of early neural induction) (Fig 3A). After the second passage, the selected cells from control, AMN and cALD IPSC were characterized by immunofluorescence for their mulitpotency. NPC were assessed for expression of neural progenitor markers such as PAX6, SOX9 and Nestin (Fig 3B–3D) and also for the expression of pluripotency markers (Oct4), as well as differentiated cell markers (β-Tubulin, CNPase) as negative markers (Fig 3E). Our data indicate excellent NPC differentiation and almost a pure cell population that is positive for the multipotency markers (Fig 3B–3E).

### Generation and characterization of neural cells (OLs, Ast and neurons)

**Fig 4:**
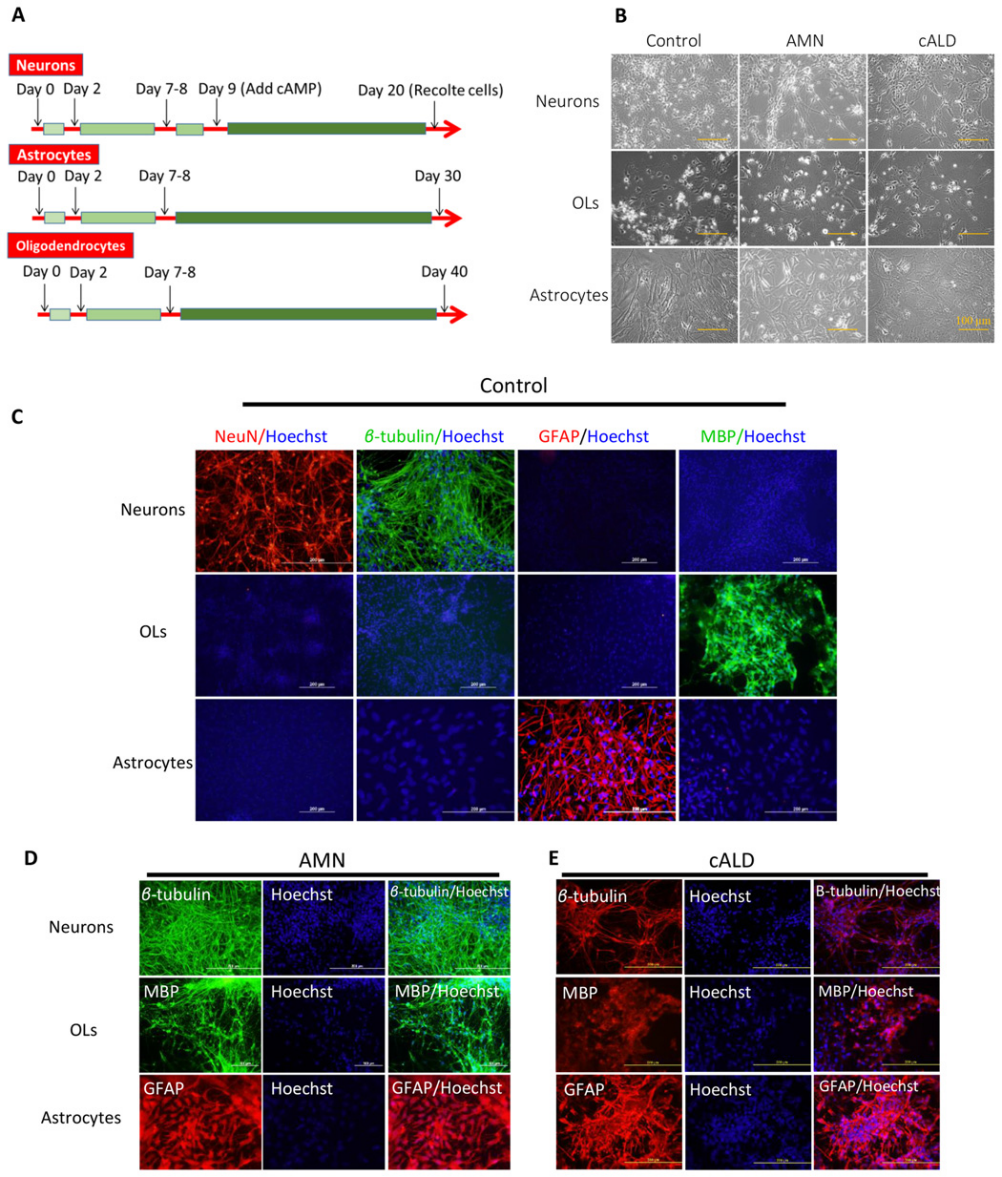
IPSC-derived brain cells differentiation and characterization. (A) Protocols for differentiation of IPSC-derived NPC into different brain cell types (neurons, Ast and OLs). (B) Morphology of different brain cell types as phase contrast images at the end of differentiation protocol, (scale bars, 100 μm). (C) Representative positive (as differentiation efficiency markers) and negative (as cell culture purity markers) immunostaining for respective markers for Neurons (NeuN and β-Tubulin as positive staining), OLs (MBP as positive staining) and Ast (GFAP as positive staining) from control cells. (D-E) Representative positive immunostaining for AMN and cALD cells for neuron markers (β-Tubulin), for Ast (GFAP) and for OLs markers (MBP).

Fig 4A shows the protocol for differentiation of NPC into neurons, Ast and OLs, NPC derived from control, AMN and cALD IPSC were cultured in neural induction media (NIM) on poly-L ornithine/laminin coated plates for 2 days and then, media was switched to the culture type specific differentiation media as described in the method section. Cells were replated if the confluency reached 80% prior to 9 days in culture. OLs were differentiated for 40 days, Ast for 30 days and neurons for 20 days. To accelerate differentiation of neurons, cAMP was added to the differentiation media at day 9 (Fig 4A). Following cell specific protocol of NPC differentiation, typical morphology of Ast, neurons or OLs, represented in Fig 4B was seen. Further characterization was performed by immunofluorescence for different cell specific markers using the respective antibodies (Fig 4C–4E). Neurons were characterized for expression of neuronal markers, (class-III β-Tubulin (Tuj1), and NeuN) as positive controls, and for GFAP and MBP as negative controls to assess the purity of differentiated neurons (Fig 4C–4E). Ast were characterized for the expression of an Ast marker, GFAP as positive control and NeuN, MBP and β-Tubulin as negative controls to assess the purity of IPSC derived Ast (Fig 4C–4E). OLs cells were characterized for the expression of OLs markers (MBP) as positive controls (Fig 4C–4E) and β-Tubulin, NeuN and GFAP as negative controls to monitor the purity of IPSC derived OLs (Fig 4C–4E). The data in Fig 4 show a protocol (Fig 4A) for NPC differentiation into OLs, Ast and neurons with purity greater than 90% (Fig 4C–4E). There is no difference between different cell types (Controls, AMN or cALD) in their potency for differentiation of NPC cells to OLs, Ast, neurons, showing no direct impact of *ABCD1* dysfunction on the ability of fibroblasts to generate IPSC and their respective neural cell types.

### Functional characterization of IPSC-derived brain cells

**Fig 5:**
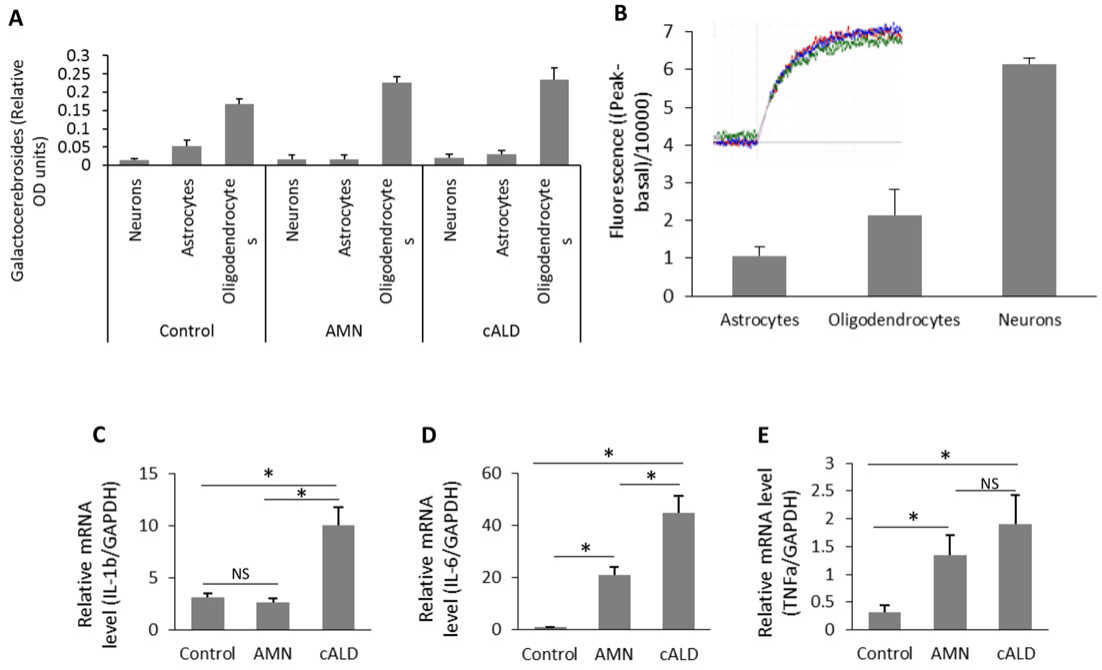
Biochemical characterization of IPSC-derived Ast, neurons and OLs. (A) Quantification of the OLs marker: galactocerebrosides purified from OLs, neurons and Ast by densitometric scanning in Control, AMN and cALD cells. (B) Quantification of the fluorescence (n = 2) related to calcium influx stimulation by glutamate in control cells (neurons, OLs and Ast). (C-D) quantification of mRNA levels of *IL-1β*, *TNFα* and *IL-6* in Control, AMN and cALD Ast by RT-qPCR (n = 3; n is the number of independent measurements from independent preparation of cells). mRNA levels were standardized with mRNA level of the *GAPDH*. Data are represented as mean±SD. *P<0.05; **P<0.01

**Fig 6:**
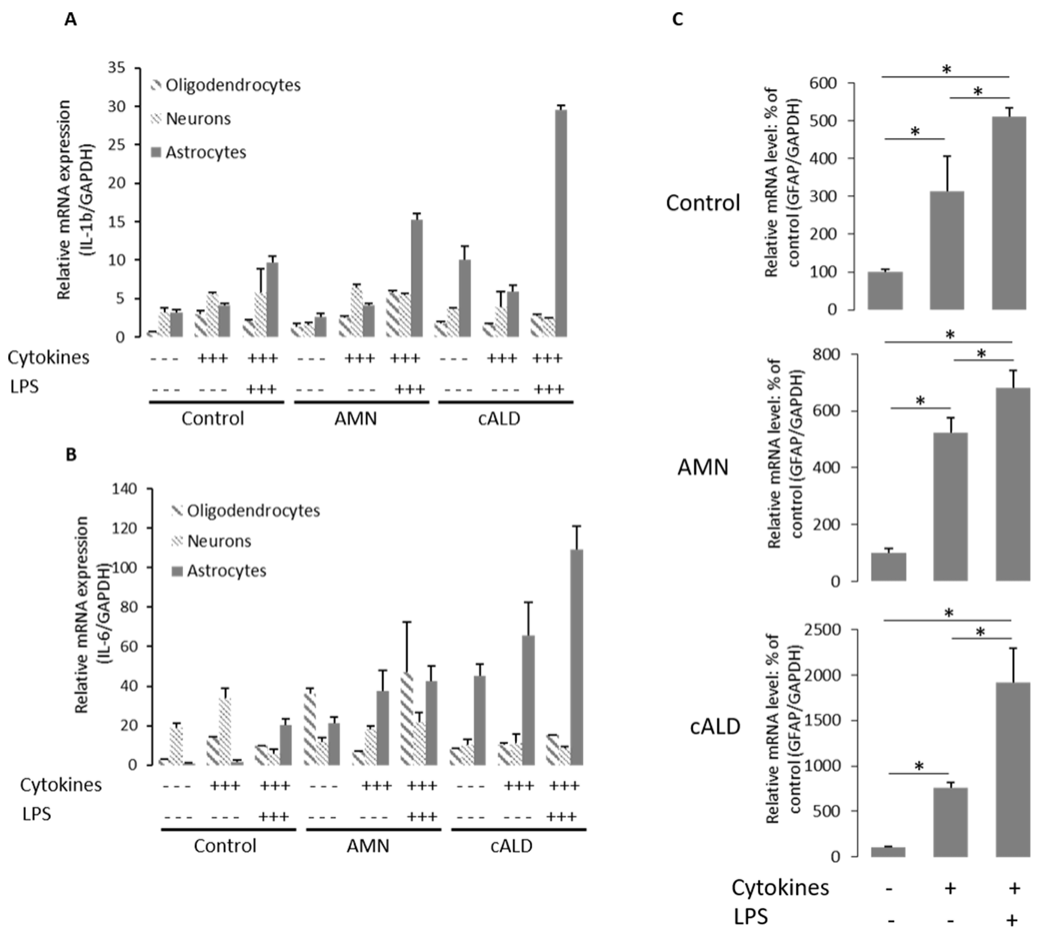
Functional characterization of different IPSC-derived brain cells. (A-B) quantification of mRNA levels of *IL-1β* and *IL-6* respectively in neurons, OLs and Ast of different cell lines (Control, AMN and cALD) by RT-qPCR (n = 2; n is the number of independent preparation of cells). mRNA levels were quantified in stimulated (cytokines or LPS with cytokines) or unstimulated cells as described in the method section. (C) Quantification of mRNA levels of *GFAP* in Control, AMN and cALD Ast treated with cytokines or LPS with cytokines by RT-qPCR (n = 3; n is the number of independent preparation of cells). mRNA levels were standardized with mRNA level of the *GAPDH*. Data are represented as % of Control mean±SD. *P<0.05.

Next, we performed functional characterization of differentiated cells (Figs 5 and 6) for cell specific functions/properties. OLs were characterized for the expression of OLs specific sphingolipid, galactocerebroside which was used as a marker for OLs [42]. Consistent with the immunofluorescence data for cell specific markers (Fig 4C–4E), the galactocerebrosides were detected only in OLs and not in neurons and Ast (Fig 5A). These data provide evidence that, MBP+/CNPase+/OSP+ cells derived from nestin+, Pax6+, and Sox9+ NPCs (Figs 4–6) harbor the properties of OLs. Neurons from IPSC-derived NPC were further functionally characterized by efflux of Ca2+ in response to glutamate stimulation (Fig 5B). As shown in Fig 5B, β-Tubulin+ neurons respond to glutamate mediated Ca2+ influx as described in the method section. Only basal activities were observed in IPSC-derived Ast and OLs, documenting the functional specificity of IPSC-derived neurons (Fig 5B). Ast are known to participate in neuroinflammatory responses in various disease conditions including cALD. Ast cultures were analyzed for their ability to produce cytokines (Figs 5 and 6). We measured the mRNA levels of different cytokines (*IL-6*, *TNFα* and *IL-1β*) in different cell types under basal (resting) status (Figs 5C–4E). There was no difference in expression of *IL-1β* mRNA between unstimulated control Ast and AMN Ast (Fig 5C). cALD Ast produced higher levels of *IL-6* and *IL-1β* mRNA under basal conditions compared to AMN (Fig 5C and 5D). A less significant increase of *IL-6* in AMN Ast than cALD Ast was observed (Fig 5D). Although the level of TNFα mRNA was higher in cALD Ast than in AMN Ast but statistically not significant (Fig 5E), indicating the cALD Ast are predisposed to inflammatory activity. Following stimulation of different cell types with cytokines (TNFα 50ng/ml, IL-1β 50ng/ml and IFNγ 100ng/ml) or cytokines plus LPS (100ng/ml) for 6h (Fig 6A and 6B). Fig 6A and 6B shows cell type dependent expression of the mRNA levels for cytokines in Ast, neurons and OLs from AMN and cALD at resting status as well as following treatment with cytokines or LPS plus cytokines. These data show that cytokines were highly expressed in Ast but not in neurons or OLs (Fig 6A and 6B). Stimulation of cALD Ast and AMN Ast with cytokines or LPS plus cytokines increased the expression of these pro-inflammatory cytokines but no such increase was observed in OLs or neurons (Fig 6A and 6B) documenting the functional specificity of IPSC-derived Ast. Under stimulatory conditions, cALD Ast expressed higher levels of mRNA for *IL-6* and *IL-1β* as compared to AMN Ast (Fig 6A and 6B). The expression levels of *GFAP* mRNA increased in response to cytokines or LPS plus cytokines treatment (Fig 6C). These studies document the purity and functional activities of IPSC cell derived brain cells with cALD, AMN disease and the controls.

### Differential accumulation of VLCFA in brain cell types derived from AMN-IPSC or cALD-IPSC

**Fig 7:**
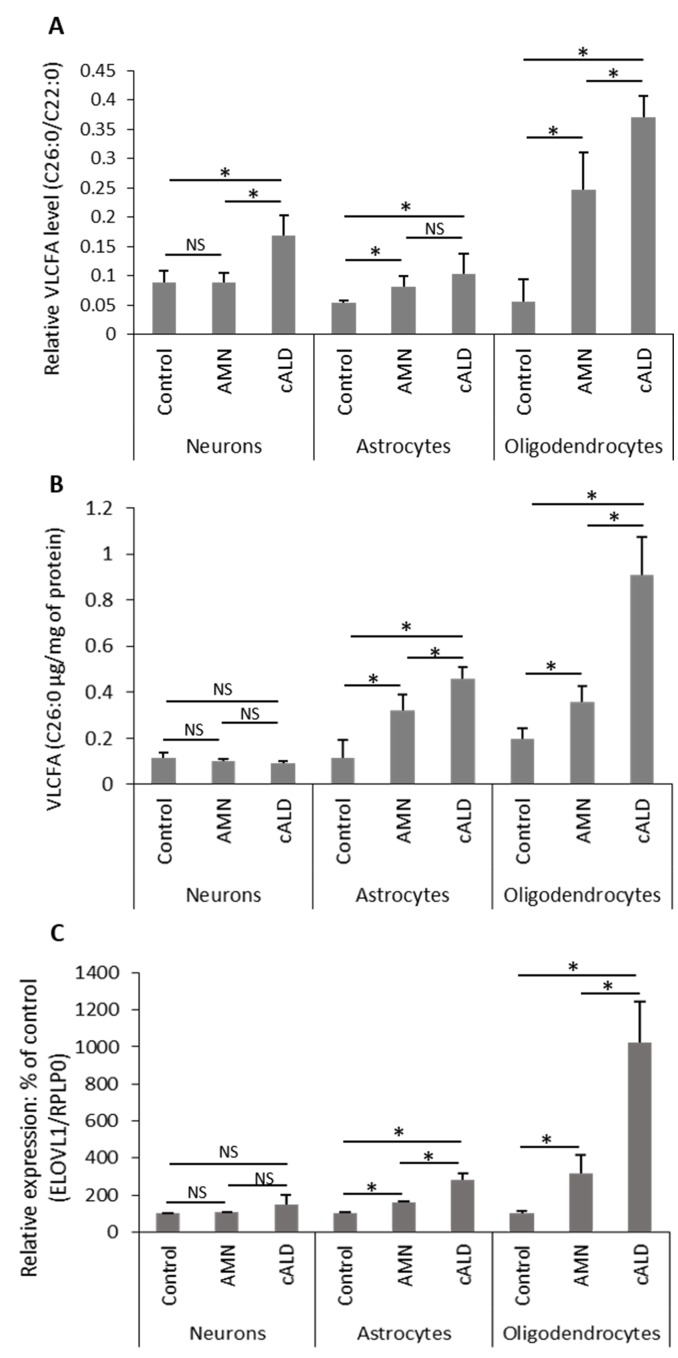
Characterization of IPSC-derived brain cells from AMN and cALD for metabolic defect. (A-B) Quantification of VLCFA (C26:0 μg/mg of protein or ratio C26:0/C22:0) in neurons, OLs and Ast from different cell lines (Control, AMN and cALD) by GC. In brief, fatty acids methyl ester was prepared directly from different cell types as described in Material and Methods. (A) VLCFA (C26:0 and C22:0) were measured as area percent of total FAs and expressed as ratio of C26:0/22:0 or (B) normalized to protein and presented as absolute amount per mg of protein in Control, AMN and cALD OLs and Ast. Results represent the means±SD from three different cell differentiation experiments; *P<0.05. (C) Quantification of mRNA levels of *ELOVL1* in different IPSC-derived brain cells from Control, AMN and cALD by RT-qPCR (n = 3); n is the number of independent preparation of cells. mRNA levels were standardized with mRNA level of the *GAPDH* or *RPLP0*. Data are represented as mean±SD. *P<0.05.

Since X-ALD disease pathology is caused by derangements in the metabolism of VLCFA resulting from deletion/mutations of *ABCD1*, we measured the levels of VLCFA in different cell types (Neurons, OLs and Ast) derived from AMN IPSC, cALD IPSC and control IPSC (Fig 7A and 7B). Saturated VLCFA (C26:0/C22:0) ratio and C26:0 levels are considered a diagnostic tool for the assessment of the peroxisomal disorders with defects in β-oxidation [8,9,17,43]. Control Neurons, Ast and OLs had almost similar levels of C26:0/C22:0 (Fig 7A). Functional loss of *ABCD1* in AMN increased the absolute levels of saturated VLCFA (C26:0 μg/mg of protein) to practically the same degree in OLs and Ast but not in neurons (Fig 7B). The levels of C26:0/C22:0 were higher in AMN-OLs than in control OLs but the highest levels were observed in cALD-OLs. Fig 7A shows a 7-fold increase in the C26∶0/C22∶0 ratio in cALD OLs as compared to the 4-fold increase in AMN OLs. The absolute amount of C26:0 in AMN OLs is increased 2 times compared to 5 times in cALD OLs. In AMN and cALD Ast, the absolute levels of C26:0 is increased and is similar to levels of C26:0 in AMN OLs (Fig 7B). Interestingly, the absolute amount of C26:0 in AMN or cALD neurons remains unchanged (Fig 7B). These observations indicate that cALD-OLs followed by Ast had highest load of VLCFA as compared to the respective AMN cells. Since cellular levels of VLCFA depend on activities for their degradation (*ABCD1*-dependent peroxisomal β-oxidation) and their biosynthesis (*ELOVL1*), we also investigated the levels of *ELOVL1* mRNA in these cell types (Fig 7C). Interestingly, cALD OLs expressed higher levels of *ELOVL1* transcripts as compared to control OLs and AMN OLs, while the increase of *ELOVL1* mRNA expression in AMN and cALD Ast was less pronounced than OLs (Fig 7C). *ELOVL1* mRNA levels in neurons from control, cALD, and AMN were comparable (Fig 7C). The levels of *ELOVL1* transcripts were 3 times higher in AMN OLs and 9 times higher in cALD OLs than control OLs and these expressions paralleled the levels of VLCFA (ratio C26:0/C22:0 and C26:0) levels in these cell types. These studies indicate the possible role of *ELOVL1* for the observed increased levels of VLCFA in OLs.

## Discussion

Lack of appropriate cell culture or animal models representing cALD and AMN diseases explains the slow scientific progress in understanding disease pathology of different phenotypes of X-ALD. This study describes that IPSC-derived brain cells (OLs, neurons and Ast) generated from skin fibroblasts from patients with cALD or AMN represent the VLCFA phenotype of cALD and AMN: 1) Accumulation of VLCFA (C26:0), but to a lesser extent in AMN OLs than in cALD OLs, consistent with previously reported levels of VLCFA in IPSC-derived AMN OLs and cALD OLs [18]; 2) Higher levels of VLCFA in cALD Ast as compared to AMN Ast; 3) Induced *ELOVL1* mRNA expression in a pattern matching the VLCFA profiles in all cell types; 4) cALD Ast compared to AMN Ast, expressed higher levels of proinflammatory cytokines (Fig 6A and 6B) and GFAP mRNAs (Fig 6C) following stimulation. These findings indicate that IPSC-derived brain cells can serve as a model to investigate the complexities of X-ALD disease. In X-ALD, excessive accumulation of VLCFA is observed in brain, adrenal cortex and testis [43,44] but due to unavailability of these cells for investigation, previous studies focused on easily accessible human cells (e.g. fibroblasts, monocytes and lymphocytes) [27,33,45,46] and rodent primary or brain cell lines [16,29,31,33,47–50]. Biochemical studies using these cells were able to document that VLCFA are oxidized in peroxisomes by the peroxisomal β-oxidation enzyme system [8] and that dysfunction/loss of *ABCD1* causes deficiency in catabolism of VLCFA [9,10,17]. Accordingly, studies using brain cell lines silenced for *Abcd1* reported accumulation of VLCFA but surprisingly a greater increase was observed in Ast cell line as compared to the expected increase in OLs cell line [16]. These cell and animal models were useful to study VLCFA metabolism but gave no insight into VLCFA derangement mediated mechanisms leading to different phenotypes of X-ALD disease. Therefore, these cell culture models have proven to be unsuitable for studying the molecular mechanisms regulating the development of different phenotypes of X-ALD. Animal models for ALD (*Abcd1* KO mice, *Abcd2* KO or *Abcd1/Abcd2* DKO) were developed soon after gene identification [19–22,34]. ALD mice express the metabolic disease but not the associated clinical neuroinflammatory disease [19–22,34]. However, older *Abcd1* KO mice do experience oxidative damage and mitochondrial dysfunction [51–55], indicating that inactivation of the *Abcd1* gene leads to late-onset neurodegenerative disease [22] and that it may represent some phenotypes of AMN [56]. We undertook a study to generate brain cells using IPSC technology, to study the disease mechanisms of different phenotypes of X-ALD (cALD vs AMN). These specific disease models provide the unique opportunity to investigate the molecular progression of the respective disease pathologies phenotypes. These models also represent a useful tool to explore the molecular differences (genetic/epigenetic) between AMN and cALD phenotypes of this disease. Therefore, cALD and AMN IPSC-brain derived cells will possibly allow the identification of markers to predict clinical progression of this disease and gain insight into the prevention of clinical disease onset. For inflammatory demyelination, MRI scanning of brains is conducted as part of the evaluation of clinically suspected patients [5]. Availability of these markers will be a major advancement in diagnosis and clinical care of the respective phenotypes of X-ALD since current diagnostic assays do not differentiate between AMN and cALD.

This study reports the morphological, immunocytochemical and functional characterization of brain cells (neurons, Ast and OLs) derived from IPSC cells generated from fibroblasts derived from patients with cALD and AMN disease. Consistent with previous studies [18,57], the loss of *ABCD1* function did not interfere in reprogramming of fibroblasts into IPSC cells for AMN or cALD (Figs 1 and 2). OLs, Ast and neurons derived from NPC were more than 80% MBP positive in OLs, 90% GFAP positive cells in Ast and 90% β-Tubulin, NeuN or neurofilament cells positive in neurons, respectively (Fig 3).

These studies provide evidence that the IPSC-derived brain cells express cell type specific functional activities, and that these cells are suitable to study the molecular mechanisms for clinical phenotypes of X-ALD disease. In addition, the studies in this manuscript describe that cALD IPSC-derived OLs and Ast for cALD and AMN express phenotype specific metabolism of VLCFA. The cALD OLs accumulated higher levels of VLCFA than AMN OLs (Fig 7A and 7B) indicating that OLs from cALD and AMN phenotype harbor cALD and AMN respective phenotypes. These observations are consistent with a previously reported study using IPSC-derived OLs, and only one study has described the generation of cALD and AMN OLs from respective IPSC [18]. The differential accumulation of saturated VLCFA (C26:0/C22:0) in these cells carry the same peroxisomal dysfunction in VLCFA suggests that in addition to the genetic defect in *ABCD1* some other mechanisms contribute to the observed differences in VLCFA load between cALD OLs and AMN OLs. Previously, we observed that silencing of *Abcd1* induced the enzyme expression for synthesis of VLCFA (*ELOVL1*) in *Abcd1* silenced OLs [16] suggesting that similar types of mechanisms may be responsible for the observed differences in VLCFA load between cALD OLs and AMN OLs. Accordingly, the *ELOVL1* mRNA expression was higher in cALD OLs than in AMN OLs (Fig 7C); similarly, cALD Ast expressed higher levels of *ELOVL1* than AMN Ast. The expression levels of *ELOVL1* mRNA paralleled VLCFA accumulation in different AMN and cALD cell types (Fig 7A and 7B). There were no differences in the mRNA levels of *ELOVL1* in cALD and AMN neurons compared to the control cell types (Fig 7C). These observations indicate that both genetic (*ABCD1* dysfunction based defect) and epigenetic (upregulation of *ELOVL1)* mechanisms contribute to the differential load of VLCFA observed in X-ALD cells. The significance of differential mRNA expression of *ELOVL1* and differential VLCFA load in cALD vs AMN OLs in disease pathology and disease phenotype is not known at present. This study reports, for the first time, the inflammatory properties of IPSC-derived AMN Ast, and cALD Ast, both under unstimulated (basal) and stimulated conditions respectively (Fig 5C–5E, Fig 6A and 6B). The higher observed expression of proinflammatory cytokines (*IL-1β* and *IL-6*) by unstimulated cALD Ast, compared to AMN Ast, suggests that cALD Ast are predisposed to a proinflammatory response. *TNFα* mRNA expression is higher in cALD Ast than in AMN Ast but is not statistically significant. IPSC-derived Ast, but not neurons or OLs produced cytokines in response to proinflammatory (cytokines or LPS + cytokines) stimulation describes the specificity of functional activity of IPSC-derived Ast (Fig 6A and 6B). The mechanism for different degrees of inflammatory response between AMN Ast and cALD Ast is not known at present, indicating that cellular mechanisms of cALD Ast are predisposed to inflammatory responses as compared to AMN Ast and control Ast (Fig 5C–5E, Fig 6A and 6B).

In summary, this study reveals that IPSC-derived neurons, Ast and OLs express cell specific activities/functions and that Ast and OLs from cALD and AMN mimic the biochemical activities of cALD and AMN (accumulation of VLCFA, inflammation) disease. Therefore, this model is an ideal platform to investigate the cell specific mechanisms regulating the epigenetic activities for expression of *ELOVL1* mRNA and VLCFA load in cALD OLs and proinflammatory responses in cALD Ast as compared to AMN Ast. In addition to the investigation of molecular mechanisms of neurodegeneration between AMN and cALD, these IPSC-derived OLs and Ast also provide a platform for testing new therapeutics for AMN vs cALD disease. Moreover, the study of these cells may identify markers for early diagnosis of different phenotypes of cALD and AMN; critical requirements for disease prognosis and patient care.

## Supporting Information

S1 Dataset This excel file has all the individual data points behind means, medians and variance measures presented in the figures of this paper. (XLSX)

## Abbreviations

X-ALD: X-linked adrenoleukodystrophy
cALD: childhood cerebral ALD
AMN: adrenomyeloneuropathy
*ABCD1*: ATP binding cassette transporter D1
ALDP: adrenoleukodystrophy protein
VLCFA: very long chain fatty acids
*ELOVL*: Elongation of very long chain fatty acids
OLs: oligodendrocytes
Ast: astrocytes
